# Survey Satisficing Inflates Stereotypical Responses in Online Experiment: The Case of Immigration Study

**DOI:** 10.3389/fpsyg.2016.01563

**Published:** 2016-10-18

**Authors:** Asako Miura, Tetsuro Kobayashi

**Affiliations:** ^1^Department of Psychological Science, Kwansei Gakuin UniversityNishinomiya, Japan; ^2^Department of Media and Communication, City University of Hong KongKowloon, Hong Kong

**Keywords:** survey satisficing, stereotyping, immigrants, online experiments, instructional manipulation check

## Abstract

Though survey satisficing, grudging cognitive efforts required to provide optimal answers in the survey response process, poses a serious threat to the validity of online experiments, a detailed explanation of the mechanism has yet to be established. Focusing on attitudes toward immigrants, we examined the mechanism by which survey satisficing distorts treatment effect estimates in online experiments. We hypothesized that satisficers would display more stereotypical responses than non-satisficers would when presented with stereotype-disconfirming information about an immigrant. Results of two experiments largely supported our hypotheses. Satisficers, whom we identified through an instructional manipulation check (IMC), processed information about immigrants' personality traits congruently with the stereotype activated by information provided about nationality. The significantly shorter vignette reading time of satisficers corroborates their time-efficient impression formation based on stereotyping. However, the shallow information processing of satisficers can be rectified by alerting them to their inattentiveness through use of a repeated IMC.

## Introduction

Though survey satisficing, grudging cognitive efforts required to provide optimal answers in the survey response process, poses a serious threat to the validity of online experiments, a detailed explanation of the mechanism has yet to be established. Therefore, focusing on attitudes toward immigrants, this study attempts to bridge the findings from social psychological research on stereotyping and those from recent research on satisficing, and elaborate the mechanism by which satisficing distorts treatment effect estimates.

### Detrimental effects of survey satisficing

Online experiments have become an increasingly common research tool; however, survey satisficing, which occurs when participants do not devote the cognitive effort required to provide optimal answers in the survey response process, poses a serious threat to the validity of this type of experiment. Studies on survey satisficing were originally developed from a concept in cognitive psychology, which is one of the cost-efficient decision-making strategies to achieve satisfactory outcomes (Simon, 1957). Because survey satisficing (“satisficing” hereafter for brevity) biases treatment effects on the dependent variables (Oppenheimer et al., 2009; Maniaci and Rogge, 2014), having many satisficers among the participants might undermine the accuracy of effect size estimates. As expected, previous research has documented that satisficing can distort results (e.g., Oppenheimer et al., 2009; Maniaci and Rogge, 2014; Berinsky et al., 2016).

Satisficing occurs when inattentive respondents give shallow responses (Couper et al., 2013; Tourangeau et al., 2013). When respondents answer a questionnaire survey, the accuracy of obtained data is dependent on their performance of required cognitive tasks. When answering, they must perform a series of mental processes to offer a valid response: (1) comprehension to interpret the intended meaning of the question; (2) retrieval to search memory for relevant information; (3) judgment to integrate retrieved information into summary judgments; and (4) response to convey the judgment (Vannete and Krosnick, 2013). Though researchers expect that all respondents always proceed carefully through each of these stages, they do not necessarily do so. Some respondents may interpret each question only superficially and select a presumably reasonable answer to each question without referring to any internal psychological cues. Other respondents may settle for generating merely satisfactory answers instead of attempting to generate an optimal answer (Krosnick, 1991).

Satisficing occurs for a variety of reasons in online experiments, including online panel members' inclination to obtain remuneration quickly (Göritz, 2004, 2006; Göritz et al., 2008) and experimenters' inability to oversee the participants during the response process (Couper, 2000). Although prior detailed studies of the potential threat satisficing poses to the validity of online experiments were primarily in the US (Maniaci and Rogge, 2014), it appears to be an international issue that also occurs in countries such as Japan (Miura and Kobayashi, 2016), Germany (Greszki et al., 2014), and Canada (Mandel, 2014).

A variety of methods to screen for satisficing have been proposed (Maniaci and Rogge, 2014), among which Oppenheimer et al.'s (2009) instructional manipulation check (IMC) is one of the most frequently used. An IMC presents typical survey items (e.g., Likert scale, check boxes) but also includes an instruction to ignore them and instead choose an atypical response to indicate that respondents are paying close attention. The original study reported that more than 30% of university students in the US failed the IMC (Oppenheimer et al., 2009), whereas other studies have revealed large variance across countries: 19% in Canada (Mandel, 2014), more than 50% in Japan (Miura and Kobayashi, 2015), and more than 70% in Spain, Mexico, and Columbia (Revilla and Ochoa, 2015); this indicates an urgent need to address the issue of satisficing in online experiments.

What is worse, there is not yet a consensus on how to handle respondents who have failed a screening test. Dropping satisficers from the sample would reduce data noise, thus increasing the study's internal validity; however, this also reduces sample diversity and can compromise the study's external validity. To avoid such a trade-off, attempts have been made to encourage satisficers to pay closer attention to the experiment by alerting them to their inattentiveness, for example, repeatedly redirecting those who have failed the IMC to the same IMC until they pass it; this resulted in the subsequent survey responses of those who initially failed the IMC aligning with the theoretically expected pattern (Oppenheimer et al., 2009). That is, Oppenheimer et al. (2009) highlighted the potential for rectifying satisficers through alerting them to their inattentiveness. In contrast, although Berinsky et al. (2016) succeeded in encouraging satisficers to pay more attention, their overall data quality did not substantially improve (see also Hauser and Schwarz, 2015).

### Satisficing and stereotyping in immigration studies

As a case for examining the mechanism by which satisficing distorts treatment effect estimates in online experiments, we focus on attitudes toward immigrants. Immigration flows are on the rise in most developed countries and related issues between migrant and native groups have led to significant tension in many parts of the world.

Against this backdrop, researchers using online experiments have produced noteworthy findings regarding attitudes toward immigrants (Iyengar et al., 2013; Hainmueller and Hopkins, 2015; Kobayashi et al., 2015). For instance, Iyengar et al. (2013) demonstrated that citizens in seven advanced industrialized democracies generally oppose more open immigration policies, but stand ready to admit individual immigrants (i.e., person positivity bias). Similarly, with a specific focus on Japan, Kobayashi et al. (2015) found that Korean immigrant workers are, *ceteris paribus*, viewed more favorably than workers from China and that affluent Japanese evaluate lower status applicants more negatively. However, the impact of satisficing needs to be more carefully examined in this specific context. Hainmueller et al. (2015) demonstrated that the design of survey experiments (i.e., vignette in which manipulations are embedded in descriptive texts and/or images vs. conjoint in which manipulations are embedded in sets of attributes) significantly influences the magnitude of treatment effects in immigration studies. More importantly, they also found evidence that satisficing mediates this influence, but a detailed explanation of the mechanism of satisficing has yet to be established in immigration studies.

In this study, we posit that satisficers are more likely to employ outgroup stereotypes than non-satisficers to form impressions of an immigrant. Building on Asch's (1946) classic study, which demonstrated that people form impressions of others in a top-down manner based on central traits such as “warm” and “cold,” Fiske and Neuberg (1990) developed a continuum model of impression formation that is now widely used among social psychologists. According to that model, the perceiver initially places the target into a social category and forms an impression of the target based on stereotypes associated with that particular category. The stereotype of the in-group tends to be positive because it helps to assert and elevate self-identity and that of the out-group tends to be negative and thus can lead to prejudice and discrimination (Tajfel and Turner, 1979). This stereotyping happens almost automatically, requiring little cognitive load (Devine, 1989).

The processes that the perceiver subsequently follows depend on whether additional information they receive is congruent with the initially activated stereotype. If this information fits the stereotype, confirmatory categorization is triggered, whereby the perceiver retains the stereotypical impression, a process that also entails little cognitive load. If, on the other hand, the information is incongruent with the stereotype and challenges the initial impression, substantial cognitive effort is required; the perceiver either recategorizes the target or, if the available information does not allow this, moves on to piecemeal integration in an attempt to understand the target not as a member of the category but as an individual person.

The implication of this model is that when forming an impression of a target, perceivers will stick to initial low-effort stereotyping if they do not have the motivation or ability to process additional counter-stereotypical information. As an illustration, Hutter and his colleagues found that the mode of processing social information is dependent on motivation and/or cognitive ability (Hutter et al., 2009, 2013). According to their findings, when a target person's social category and stereotype are congruent (e.g., a female nurse), perceivers can form impressions swiftly with little effort, whereas it takes greater time and cognitive effort to form impressions when the target person has a counter-stereotypical trait (e.g., a female mechanic). In relation to the survey response process, satisficers, who tend to begrudge the cognitive efforts required to provide optimal answers, will find it harder to move on to recategorization or piecemeal integration when faced with additional counter-stereotypical information, leading to more stereotypical responses.

Based on the above arguments, we posit the following hypotheses.

Hypothesis 1. When participants are provided with both a target's nationality to activate stereotyping and a personality trait that is congruent with this national stereotype [positive central trait for the in-group target (warm), negative central trait for the out-group target (cold)], initial categorization will be confirmed and participants will form a stereotypical impression. Because this categorization requires little cognitive effort, satisficing will not affect the response; that is, regardless of the level of satisficing, all participants will form a positive impression of the in-group target and a negative impression of the out-group target.
Hypothesis 2. When participants are provided with both a target's nationality to activate stereotyping and a personality personal trait that is incongruent with this national stereotype [negative central trait for the in-group target (cold), positive central trait for the out-group target (warm)], they will proceed to either recategorization of the target or piecemeal integration before making an individuated assessment. Because recategorization and piecemeal processing require considerable cognitive effort, compliers, who do not show any satisficing behavior, will invest the cognitive resources necessary to process the stereotypically incongruent trait information and update their initial stereotypical impression, leading to an impression that is inconsistent with the stereotype. In contrast, satisficers will not invest the cognitive resources necessary to process the stereotypically incongruent trait information and thus will form an impression that is consistent with the initially activated stereotype. That is, satisficers will form more positive impressions of the in-group target than will compliers (Hypothesis 2a) and satisficers will form more negative impressions of the out-group target than will compliers (Hypothesis 2b).

We tested these hypotheses in the context of Japanese people's sentiments toward Chinese immigrants. Even though Chinese people share a number of similar physical features with the Japanese and Western people tend to apply similar stereotypes to Chinese and Japanese (Madon et al., 2001), there is a strong sense of “ethnic” distinctiveness at least from the Japanese perspective (Tsukamoto and Karasawa, 2015). The Japanese sentiment toward Chinese has become increasingly negative in recent years despite the number of Chinese people living in Japan having trebled in the past 20 years (Ministry of Justice Government of Japan, 2015). According to the Cabinet Administration Office Government of Japan (2014), only 14.8% of Japanese people feel an affinity toward China, a figure that is remarkably low compared to that felt toward other countries, such as South Korea (31.5%) and the United States (82.6%). Given the prevalence of satisficing in online research (Miura and Kobayashi, 2015) and the strong negative stereotype held toward Chinese people (Akuto and Hara, 2000; Kamise et al., 2010; Kobayashi et al., 2015), Japan offers a suitable context for testing our hypotheses.

## Study 1

### Methods

We employed a vignette-style online survey experiment in which the participants formed impressions of a hypothetical immigrant. Impression of an individual immigrant is regarded as an operational definition of the attitude toward immigrants. In other words, we employed participants' impressions of the Chinese target as an index of attitudes toward Chinese immigrants in Japan. This type of online experiment is becoming increasingly common in immigration studies (e.g., Aalberg et al., 2012; Iyengar et al., 2013; Kobayashi et al., 2015). At the same time, we also examined whether we can minimize the detrimental effect of satisficing by alerting satisficers to their inattentiveness.

#### Participants

The experiment was conducted from May 13–19, 2015, with participants recruited from the online panel of a leading Japanese survey firm (Nikkei Research Inc.) via email. Those who completed the experiment received lottery-based remuneration. Of 40,900 potential participants who were solicited for the study, 5389 accepted, and 4693 completed the experiment. Though the response rate is admittedly low, it is not uncommon in online recruitment of participants. For instance, when we recruit participants through Amazon Mechanical Turk, a far greater number of Turkers are exposed to the recruitment message than those who actually participate in the study. The low response rate in our study is analogous to this situation. We can only send soliciting e-mails to potential participants, but we do not even know whether they read it. Forty-two participants were excluded from the following analyses because they took more than 1 h to finish the experiment. The mean age of the participants was 49.41 years (*SD* = 13.86) and 58.2% were male.

#### Experimental design and measurements

First, we measured demographic variables, the device used to respond to the survey, and the environment in which responses were provided (e.g., at home), as well as two covariates that predict satisficing: frequency of participation in surveys (Whitsett, 2013) and need for cognition (NFC; Oppenheimer et al., 2009; see Supplementary Materials for detailed information about the measures).

Next, we presented an IMC similar to the one used by Oppenheimer et al. (2009) (see Supplementary Image 1 in Supplementary Materials for a translation of the original Japanese version). This began with the heading “This is a question about your everyday behavior,” which was followed by 384 characters and 8 sentences of instructions in the original Japanese version. Embedded in the latter portion of the text was the following instruction: “Please select “Yes” and then click “>>” to proceed to the next page.” The question item to which this instruction refers is “I have never used e-mail,” below which three options were presented: “Yes,” “No,” and “Don't know.” Because all participants were recruited via e-mail, the “yes” option was counterintuitive unless they carefully read the instruction. Participants who selected “yes” passed the IMC, whereas those who selected “No” or “Don't know” failed (that is, satisficed) and were redirected back to the same IMC; however, in this presentation, the embedded instruction “Please select “Yes” and then click “>>” to proceed to the next page” was highlighted in red for emphasis. Repeating the IMC was intended to make participants who failed the first check more conscious of their inattentiveness. The IMC was repeated twice at the most, and participants who failed both times proceeded to the subsequent experiment without receiving further alerts.

After completing the IMC, the participants proceeded to the impression formation experiment. Before presenting the vignette, we measured feeling thermometer scores of Japanese and Chinese people as covariates (range: 0–100). The presentation order was randomized.

**Figure 1:**
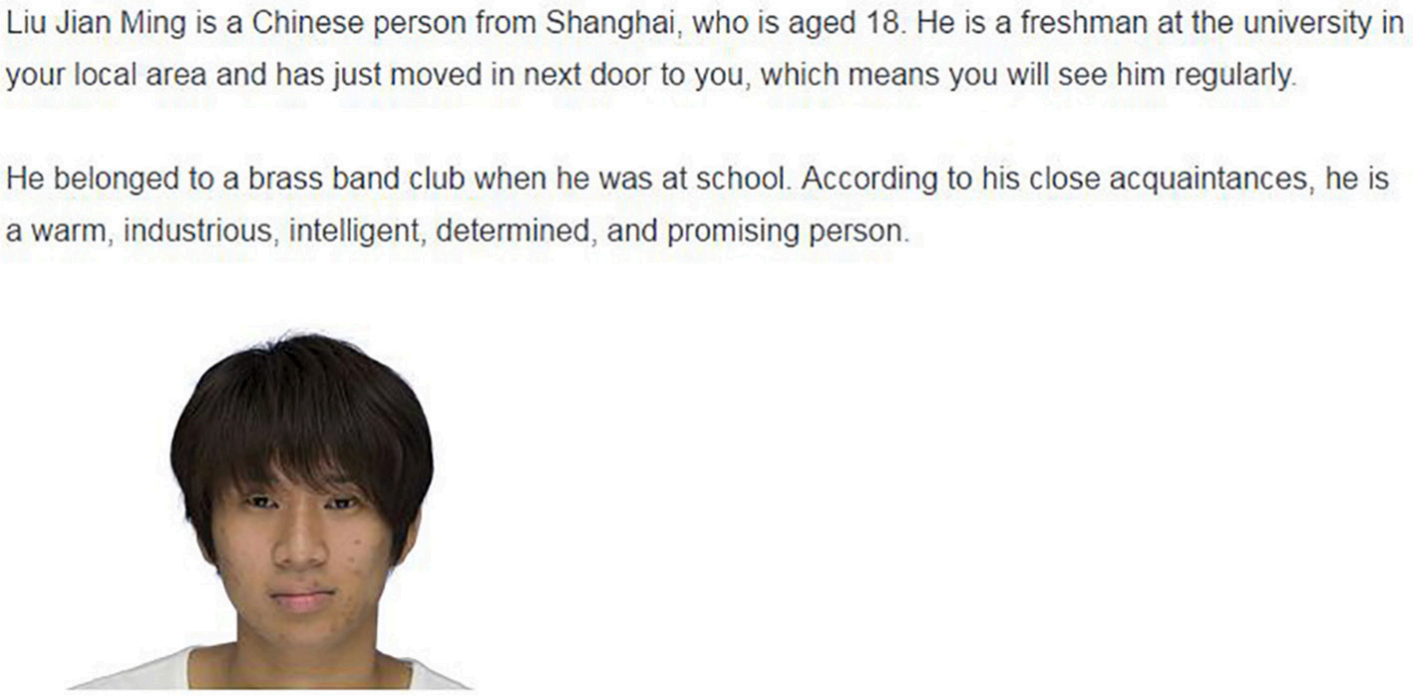
**Sample vignette of Study 1 (Nationality: Chinese, Trait: Warm)**.

Subsequent to the measurement of feeling thermometer scores, participants were randomly assigned to one of six conditions. The vignette presented to participants included a profile of the hypothetical target person and his photograph (see Figure 1 for a translation of the original Japanese version). The target person was described as an 18-year-old man who had moved to the participant's neighborhood to enroll at a university. We manipulated the target's nationality—they were described as being either Japanese or Chinese with a relevant name and birth place (e.g., Japanese/Chinese name and Aichi/Shanghai)—and personality traits by setting three conditions: warm, cold, and control (no central trait). Central traits were embedded in the description of the target's personality, along with four other adjectives (energetic, intellectual, decisive, and promising). In summary, the experiment had a 2 (nationality) × 3 (central traits) full-factorial between-subjects design. We also measured the time spent on reading the vignette. Reading time is defined as the duration between the time when the vignette was presented and the time when the participants clicked the button to move on to the next page (i.e., the total amount of time the participants stayed on the vignette on the page). Note that, as researchers cannot monitor the participants, unlike in a lab experiment, any sort of distraction such as e-mail checking, phone calls, and people in the same room, may have interrupted their reading. Therefore, the analysis of reading time is positioned as auxiliary.

After reading the vignette, participants' impressions of the target person were measured as a dependent variable using nine semantic differential items rated on a 5-point scale. This procedure is consistent with Asch (1946) and included bipolar rating adjectives (see Supplementary Table 1 for an English translation of the original Japanese version).

### Results

#### Satisficing (IMC)

We classified participants who passed the first IMC (57.7%) as compliers, those who failed the first but passed the second IMC (32.3%) as converts, and those who failed both IMCs (10.0%) as satisficers. An ordered logistic regression predicting the level of satisficing (compliers = 0, converts = 1, satisficers = 2) indicated a positive effect of frequency of survey participation (B = 0.15, *p* < 0.01) and a negative effect of NFC (B = −1.09, *p* < 0.01), which is consistent with findings of Study 1 by Oppenheimer et al. (2009) (see Supplementary Table 2).

As for attrition during the experiments, we identified at which stage they dropped out from the studies. Of all 696 participants who did not complete the experiments, 471 participants stopped responding to the questionnaire before the IMC was presented and thus their satisficing level is unknown. Satisficers were more likely to drop out than compliers (7.9 vs. 3.7%), and converts (5.2%) were intermediate. This is not surprising because satisficers are, by definition, not sufficiently motivated and thus are more likely to lose interest in the study. Most importantly, the level of satisficing was well-balanced across six conditions (Chi-square = 5.72, *df* = 10, *p* = 0.84). Therefore, although there was significant attrition and satisficers were more likely to drop out, this did not impair random assignment, and thus, the estimated treatment effect was still unbiased.

#### The effect of satisficing on impression formation

**Figure 2:**
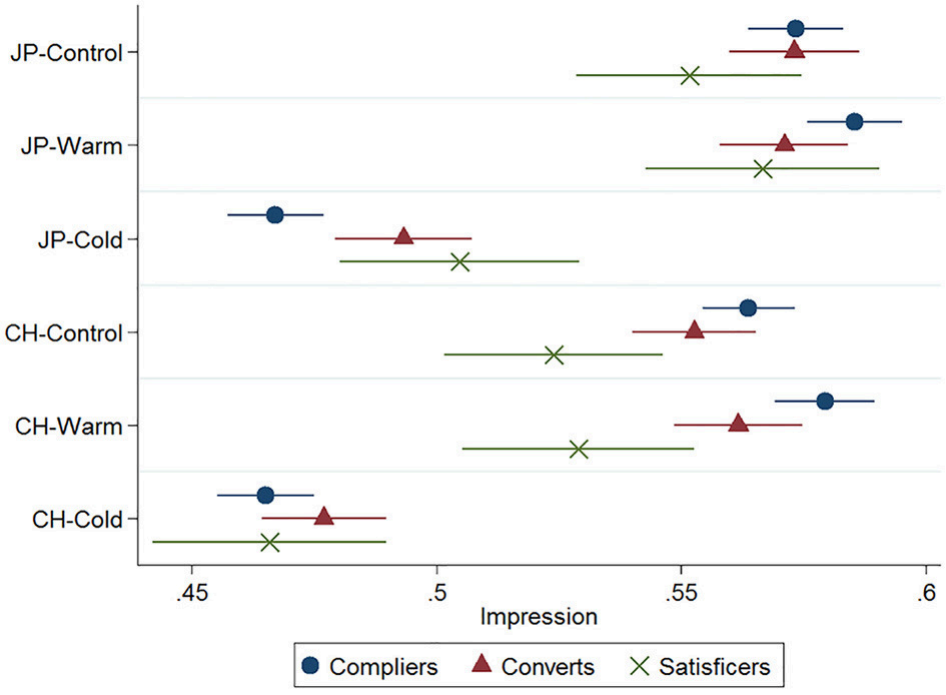
**Point estimates on impression of the target with 95% CI (Study 1; JP, Japanese; CH, Chinese)**.

We estimated a multiple regression model in which the dependent variable was impression of the target and the independent variables were the two treatments (nationality and central trait), level of satisficing (compliers/converts/satisficers), and the interaction terms between them (Table 1). To increase the efficiency of estimation, we included participants' feeling thermometer scores for Japanese and Chinese people as covariates. Figure 2 shows the point estimates and their 95% confidence intervals (CI) from a linear model fitted by ordinary least squares.

**Table 1:** **Multiple regression models predicting the impression of the target**.

**DV: Impression of the target person**		**Study 1**	**Study 2**
		**Coef. (B)**	
Nationality (Baseline: Japanese)	Chinese	−0.010	−0.009
		(0.007)	(0.013)
Trait (Baseline: Control)	Warm	0.012+	0.075^**^
		(0.007)	(0.013)
	Cold	−0.106^**^	−0.044^**^
		(0.007)	(0.013)
Nationality × Trait (two-way)	Chinese × Warm	0.004	−0.007
		(0.010)	(0.019)
	Chinese × Cold	0.008	0.001
		(0.010)	(0.019)
Satisficing level (Baseline: Compliers)	Converts	−0.000	0.010
		(0.008)	(0.015)
	Satisficers	−0.022+	0.011
		(0.013)	(0.020)
Nationality × Satisficing level (two-way)	Chinese × Converts	−0.011	−0.007
		(0.012)	(0.021)
	Chinese × Satisficers	−0.018	−0.005
		(0.018)	(0.031)
Trait × Satisficing level (two-way)	Warm × Converts	−0.014	−0.026
		(0.012)	(0.023)
	Warm × Satisficers	0.003	−0.034
		(0.018)	(0.031)
	Cold × Converts	0.026^*^	−0.005
		(0.012)	(0.021)
	Cold × Satisficers	0.059^**^	−0.039
		(0.018)	(0.031)
Nationality × Trait × Satisficing level (three-way)	Chinese × Warm × Converts	0.008	0.004
		(0.017)	(0.031)
	Chinese × Warm × Satisficers	−0.013	−0.050
		(0.026)	(0.047)
	Chinese × Cold × Converts	−0.003	−0.025
		(0.017)	(0.030)
	Chinese × Cold × Satisficers	−0.019	−0.021
		(0.026)	(0.047)
Covariates	Feeling thermometer (Chinese)	0.001^**^	0.001^**^
		(0.000)	(0.000)
	Feeling thermometer (Japanese)	0.001^**^	0.001^**^
		(0.000)	(0.000)
Constant		0.495^**^	0.425^**^
		(0.009)	(0.016)
Number of observations		4651	1309
*R*^2^		0.195	0.204

Standard errors in parentheses.

** *p < 0.01*,

* p < 0.05,+ p < 0.1.

Consistent with the assumed negative stereotype against Chinese people, the mean impression score in the Chinese nationality condition was lower than the Japanese nationality condition by 0.014 points (*p* < 0.01). When the target's trait information conformed to the national stereotype, that is, under Chinese-Cold (“CH-Cold”) and Japanese-Warm (“JP-Warm”) conditions, the impression scores of compliers and satisficers were statistically indistinguishable. This finding supports Hypothesis 1. Participants who were provided with trait information that was consistent with the national stereotype confirmed their initially activated stereotype. Because this process entailed minimal cognitive effort, the responses were not affected by satisficing.

In contrast, the responses were affected by the level of satisficing when the trait information and national stereotype were incongruent. As shown in Figure 2, under the CH-Warm condition, compliers formed a significantly more favorable impression than satisficers did, whereas under the JP-Cold condition, they formed a significantly less favorable impression than satisficers did. This finding supports Hypotheses 2a and 2b, in that satisficers were less motivated in the response process; thus, when presented with stereotype-incongruent personal trait information, they failed to switch to the more cognitively demanding processes of recategorization or piecemeal integration. As a result, they were more likely to retain the initially activated stereotypical impression. On the other hand, converts and compliers showed few differences, the exception being that compliers gave significantly less favorable scores than converts did under the JP-Cold condition. This suggests that those participants who initially failed the IMC but subsequently passed it after receiving instructive feedback employed enough cognitive effort to duly consider the incongruent information.

It should be noted that, contrary to our expectation that the overall mean scores of the control condition would be between those of the warm and cold conditions, the impression scores in the control condition were quite similar to those under the warm condition. This result may be attributable to the fact that the traits given in addition to the central trait were all positive. We address this issue in Study 2.

#### Auxiliary analysis of reading time

Next, to corroborate that satisficers who were identified with IMC actually paid less attention to the vignette, we estimated a multiple regression model predicting the reading time. Reading time was logarithmically transformed to address its skewed distribution. The collection of independent variables was identical to that shown in Table 1 (see Supplementary Table 3 and Supplementary Image 2 for the estimated model, point estimates, and their 95% CI).

Reading time was longer in the Japanese than Chinese nationality condition (*p* < 0.01). Because the Chinese target was an out-group member for Japanese participants, it was easier for them to form an impression based on stereotypes. The lower cognitive effort requirement in stereotypical impression formation explains the shorter viewing time in the Chinese nationality condition. Furthermore, viewing time of converts and satisficers was significantly shorter than that of compliers, which corroborates the theoretical prediction that satisficers do not effortfully process the information presented in the vignette. It should be noted, however, that converts' viewing time was longer than that of satisficers and closer to that of compliers. This finding indicates that converts devoted more cognitive effort to examining incongruent information, suggesting the effectiveness of rectifying satisficers.

### Discussion

Study 1 demonstrated that when the information presented in the vignette was consistent with stereotypes, the responses of compliers and satisficers were indistinguishable, presumably because minimal cognitive effort was required when forming stereotypical impressions (Macrae et al., 1994). On the other hand, when the information was incongruent with stereotypes, the responses of compliers and satisficers were substantially different, arguably because processing stereotypically incongruent information entailed a substantial cognitive load (Garcia-Marques and Mackie, 1999). Analysis of reading time supported this interpretation: satisficers spent less time reading the vignette compared to compliers, suggesting low motivation and shallow information processing. These findings implied that, if the sample had a large proportion of satisficers and the presented experimental stimulus entailed cognitive load, the average impression of the immigrant would be unduly negative.

A notable limitation of Study 1 was the suboptimal setting of the control condition, in which the included traits presented a positive, rather than neutral, picture of the target; this could explain why the results under the control and warm conditions were similar. To address this issue as well as to verify the robustness of the findings from Study 1, we conducted a second study, wherein all personal trait information was removed from the control condition vignette to more deeply probe the impact of satisficing on the process of impression formation.

According to Darley and Gross (1983), stereotype-activating social labels (e.g., nationality) do not directly shape the impression of the target, but do indirectly bias the evaluation by prompting the perceiver to process additional information consistent with the activated stereotype. Therefore, when the perceiver has no information about the target other than nationality, the activated stereotype will not influence the impression of the target, but it will be influential when additional information about the target's personal traits is available because it will be interpreted consistently with the activated stereotype. Similarly, Brewer (1996) maintains that category-based impressions *increase* along with a certain amount of individuating information and attain the highest level when a moderate amount of individuating information is available to bolster judgments. In light of these findings, stereotypical impression formation based on category information (i.e., nationality) is predicted to be largely muted when only trivial information describing the target person is provided in the control condition. In contrast, stereotypes will come into play when a greater amount of individuating information regarding his personality, including warm/cold evaluation by peers, is provided in the treatment conditions, making the vignette more diagnostic than that of the control condition.

In relation to satisficing, we predict that, unlike in Study 1, the impressions formed by compliers and satisficers would be indistinguishable in the control condition. On the other hand, we predict that the responses of satisficers and compliers would be significantly different when stereotypically incongruent trait information was provided, which is essentially a replication of Study 1. Verifying these predictions will give more credence to the argument that what drives satisficers' apparent in-group favoritism is not the nationality of immigrants, but, rather, their stereotypical and low-effort processing of subsequent trait information.

## Study 2

### Methods

#### Participants

The experiment was conducted from December 9–15, 2015. Participants were recruited in the same way as in Study 1, with those who had participated in Study 1 being excluded. Of 17,341 potential participants who were solicited for the study, 1709 accepted and 1320 completed the experiment. Response and completion rates were roughly the same as in Study 1. Ten participants were excluded from the following analyses because they took more than 1 h to finish the experiment. The mean age of the participants was 49.28 years (*SD* = 12.83) and 56.8% were male.

#### Experimental design and measurements

**Figure 3:**
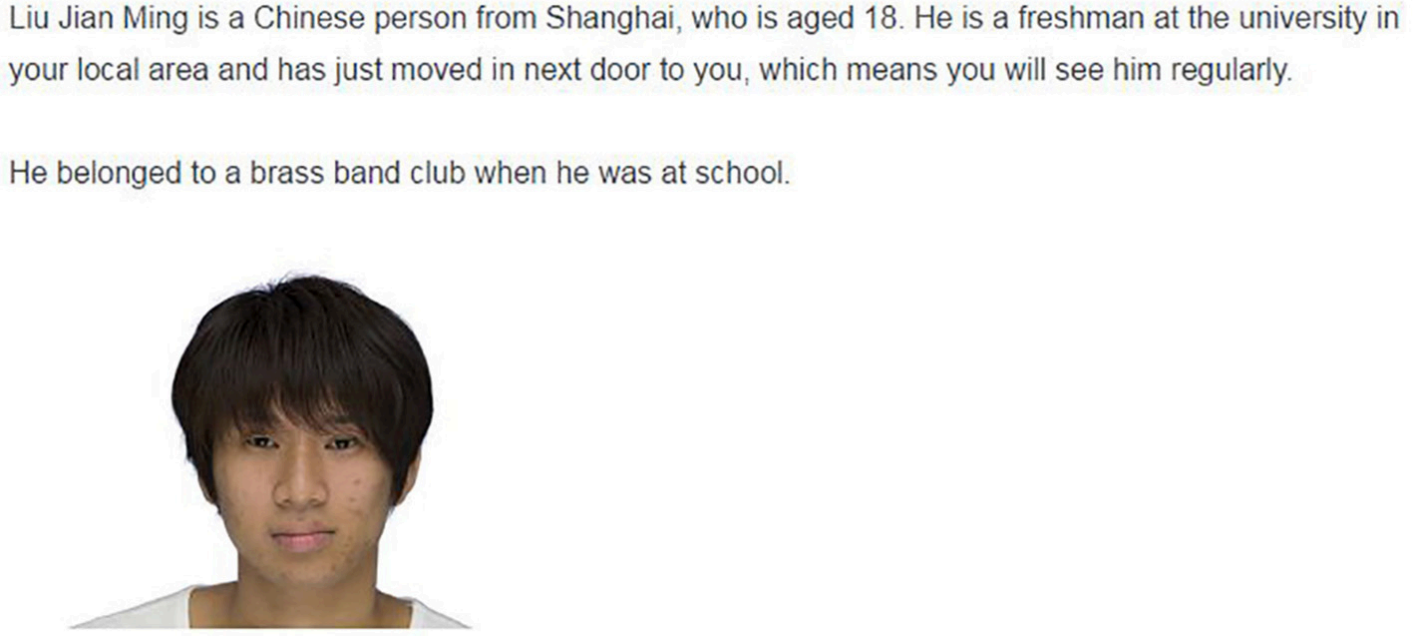
**Sample vignette of Study 2 (Nationality: Chinese, Trait: Control)**.

The experimental design was the same as in Study 1, except for the control condition vignette, in which all personality trait information was removed (see Figure 3 for a translation of the original Japanese version). Trait information in the warm and cold conditions was the same as in Study 1.

### Results

#### Satisficing (IMC)

Among the participants, 55.9% passed the IMC the first time (compliers), 33.6% failed the first but passed the second IMC (converts), and 10.6% failed both IMCs (satisficers). An ordered logistic regression predicting the level of satisficing (compliers = 0, converts = 1, satisficers = 2) indicated a positive effect of frequency of survey participation (B = 0.20, *p* < 0.01) and a negative effect of NFC (B = −0.66, *p* < 0.10), which is consistent with Study 1 (see Supplementary Table 2).

As for attrition during the experiments, we identified at which stage they dropped out from the studies and obtained almost the same results as in Study 1. Satisficers were more likely to drop out than compliers (7.3 vs. 3.6%), while converts (6.4%) were intermediate. The level of satisficing was balanced across six conditions (Chi-square = 7.79, *df* = 10, *p* = 0.65).

#### The effect of satisficing on impression formation

**Figure 4:**
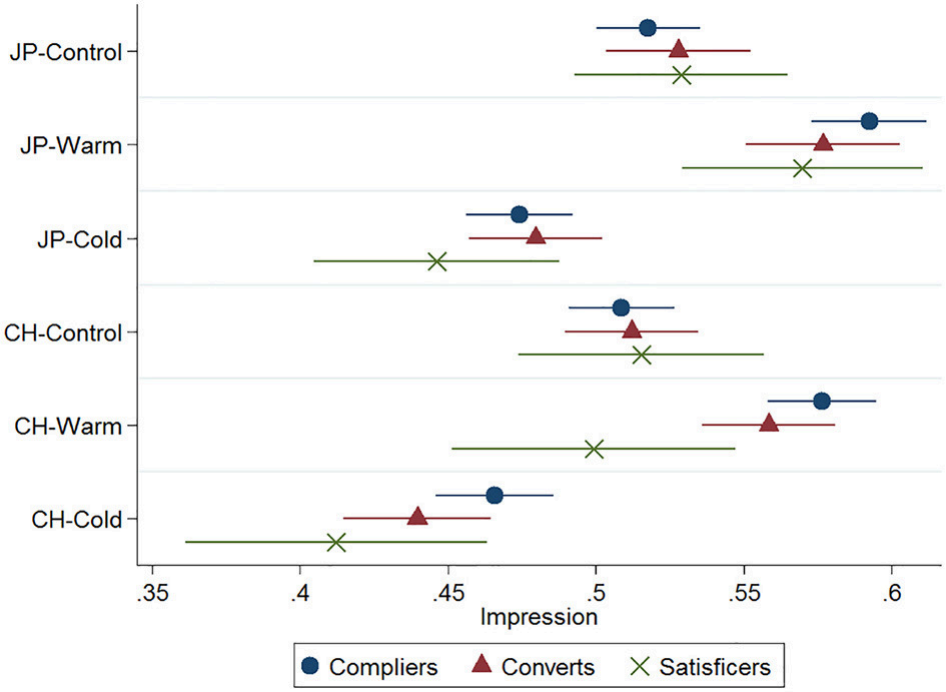
**Point estimates on impression of the target with 95% CI (Study 2; JP, Japanese; CH, Chinese)**.

We estimated the same multiple regression model as in Study 1 (Table 1). Figure 4 shows the point estimates and their 95% CI from a linear model fitted by ordinary least squares.

Unlike in Study 1, the mean impression scores of compliers and satisficers did not differ under the control conditions; this suggested that removing all of the personality trait information successfully neutralized the impression of the target. Further, the mean scores for CH-Control and JP-Control conditions were not statistically distinguishable, suggesting that nationality information did not shape participants' negative impressions, consistent with Darley and Gross (1983) and Brewer (1996).

The mean impression score in the Chinese nationality condition was lower than in the Japanese nationality condition by 0.009 points (*p* = 0.128). Though this difference was not statistically significant, the direction of the effect was consistent with Study 1. When the target's trait information conformed to the national stereotype, that is, under CH-Cold and JP-Warm conditions, the impression scores of compliers and satisficers were statistically indistinguishable. This finding replicated that of Study 1 and supported Hypothesis 1.

Under the CH-Warm condition, compliers formed a significantly more favorable impression than satisficers did, which replicated the finding of Study 1 and supported Hypothesis 2b. However, unlike Study 1, the responses of compliers and satisficers were not statistically distinguishable under the JP-Cold condition, which did not support Hypothesis 2a. Therefore, the findings of Study 1 were partially replicated.

As shown in Figure 4, the converts' pattern of responses was not significantly distinguishable from that of compliers suggesting that satisficers who were alerted to their inattentiveness in the repeated IMC changed their attitude and subsequently processed the information in vignettes at the same depth as compliers did. The consistent findings we obtained across these two studies give credence to the effectiveness of the IMC-based alert in calling attention to the experimental materials.

#### Auxiliary analysis of reading time

To support the argument that satisficers paid less attention to the vignette, we estimated the same multiple regression model as in Study 1 (see Supplementary Table 3 and Supplementary Image 3 for the estimated model, point estimates, and their 95% CI). Consistent with Study 1, reading time was significantly longer under the Japanese than Chinese nationality condition (*p* < 0.01) and reading time of satisficers was significantly shorter than compliers (*p* < 0.01). However, converts' reading time was significantly shorter than compliers in only 1 out of 6 conditions, suggesting that converts processed the information in the vignette at the same depth as compliers did.

### Discussion

With the aim of elaborating the process by which satisficers process experimental stimuli in a stereotypical way, Study 2 was designed to replicate Study 1 with an improved control condition that was neutral in terms of personality traits. Despite the smaller sample size, the findings of Study 1 were partially replicated especially in the outgroup member with a stereotype-incongruent trait (Chinese-Warm) condition, giving credence to their robustness.

Under the control condition, impressions were neutral regardless of the nationality of the target and the level of satisficing. This corroborates the findings of Darley and Gross (1983) that a stereotype-activating social label (i.e., nationality) merely stimulates expectancy; it is only when additional information (i.e., personality) is provided that the resulting impression becomes stereotypically biased. It should be noted, however, that satisficers formed significantly more negative impressions than compliers did under the CH-Warm condition, but did not form more positive impressions under the JP-Cold condition, which is inconsistent with Study 1. That is, satisficers' in-group-favoring response when provided with stereotype-incongruent information was observed only when the target was an out-group member. This asymmetry is presumably related to the imbalanced cognitive resources people use when processing information about out-groups vs. in-groups (e.g., Rodin, 1987; Sporer, 2001). Because social exchanges usually take place among in-group members, the return on effortful processing of out-group information is relatively small, which could have led to shallower processing under the Chinese than Japanese condition when stereotype-incongruent information was provided. However, given the inconsistency in the results of Studies 1 and 2, further research is needed to test the robustness of this finding.

## General discussion

Focusing on attitudes toward immigrants, we examined the mechanism by which survey satisficing distorts treatment effect estimates in online experiments. We hypothesized that satisficers would display more stereotypical responses than compliers did when presented with stereotype-disconfirming information about an immigrant. Results of two experiments largely supported the hypothesis with some notable inconsistencies. Satisficers identified through IMC processed information about the personality of immigrants in a way that was congruent with the stereotype, which was activated by the information about nationality. The significantly shorter vignette reading time of satisficers corroborates their time-efficient impression formation based on stereotyping. These results are in line with Hutter et al.'s works that when faced with an incongruent profile of a target person, those with higher motivation (Hutter et al., 2009) and those who engage in deeper processing (Hutter et al., 2013) can switch more flexibly from category-based to an individuated mode of processing. Furthermore, our results suggested that the shallow information processing of satisficers can be rectified by alerting them to their inattentiveness through use of a repeated IMC.

In studying attitudes toward immigrants, an increasing number of studies have been employing online experiments with a vignette-style factorial design or conjoint analysis (Iyengar et al., 2013; Hainmueller and Hopkins, 2015). These experiments are producing invaluable findings, but the cognitive load on participants is relatively high; they need to carefully examine the information in vignettes or repeatedly compare the detailed attributes of two immigrants. Given the low motivation of satisficers, it cannot be assumed that all participants use enough cognitive effort to process the experimental stimuli. If, as we demonstrated in this study, satisficers tend to form more stereotypical impressions of immigrants than compliers do, the racial in-group favoritism exhibited in immigration studies can be, to some extent, explained by the number of satisficers in the sample.

In this regard, we found that the responses of converts and compliers were largely indistinguishable, suggesting that satisficers who received an attention reminder after being alerted to their low motivation processed subsequent experimental stimuli with due depth. Therefore, using a repeated IMC is useful to minimize the number of satisficers in a subsequent experiment.

There are a few notable limitations in this study. First, the IMC used in this study was different from the original one. Participants were expected to give an answer known to be false whereas the original IMC asked participants to skip questions, or click on a dot hidden on the screen. We cannot rule out the possibility that such differences influenced the results. For instance, our version of the IMC might have screened out those who find it difficult to reconcile conflicting information, rather than unmotivated satisficers, which might explain why they were not good at integrating counter-stereotypical information to form impressions. However, if satisficers are people who find it difficult to reconcile conflicting information, they would have tried to process it deeply and highly conscientiously to resolve the confusion, thus taking a longer time than compliers. However, the analysis on the time spent on responding to the IMC and reading the vignette indicates satisficers spent a significantly shorter time than compliers. This strongly suggests that, instead of “struggling” with the conflicting information, “satisficing” due to low motivation was at work. With that said, replication with the original version of the IMC is desirable to rule out such alternative explanation.

Another notable limitation is that the regression model demonstrated few significant effects and the coefficients of the Trait × Satisficing level Interaction showed non-significant but negative effects in Study 2, which is inconsistent with Study 1. That is, satisficers did not form more positive impressions than compliers under the JP-Cold condition as opposed to the predicted result, although they formed significantly more negative impressions under the CH-Warm condition, which is consistent across Study 1 and 2. We suspect this inconsistency is due to the sampling variability and limited number of satisficers in Study 2. To enhance ecological validity, this study observed the participants' level of satisficing using the IMC instead of experimentally manipulating it. As a consequence, the number of satisficers was small especially in Study 2, resulting in the wide CIs shown in Figure 4. The large uncertainty surrounding the point estimates of satisficers prevents us from obtaining consistent and conclusive evidence in this regard. To further validate our hypotheses beyond the two experiments in this study, it would be ideal to field a study in which level of satisficing is experimentally manipulated so that the number of satisficers becomes comparable to that of compliers.

## Ethics statement

The research was reviewed and approved by the Kwansei Gakuin University Institutional Review Board for Behavioral Research with Human Participants (approval number 2014-39). When the research was performed, we complied with their regulations.

## Author contributions

Both authors contributed to the development of the study concept and design, and the data analysis and interpretation. AM drafted the manuscript, and TK provided critical revisions. Both authors approved the final version of the manuscript for submission.

### Conflict of interest statement

The authors declare that the research was conducted in the absence of any commercial or financial relationships that could be construed as a potential conflict of interest.

## References

[B1] Aalberg T. Iyengar S. Messing S. (2012). Who is a ‘deserving’ immigrant? An experimental study of Norwegian attitudes. Scand. Pol. Stud. 35, 97–116. 10.1111/j.1467-9477.2011.00280.x

[B2] Akuto H. Hara Y. (2000). How do people form their impressions of partner countries? From the results of public opinion research in Japan, Korea, and China (2nd report). NHK Monthly Report Broadcast Res. 50, 56–93 (in Japanese).

[B3] Asch S. E. (1946). Forming impressions of personality. J. Abnorm. Soc. Psychol. 41, 258–290. 10.1037/h0055756 20995551

[B4] Berinsky A. J. Margolis M. F. Sances M. W. (2016). Can we turn shirkers into workers? J. Exp. Soc. Psychol. 66, 20–28. 10.1016/j.jesp.2015.09.010

[B5] Brewer M. B. (1996). When stereotypes lead to stereotyping: the use of stereotypes in person perception: the use of stereotypes in person perception, in Stereotypes and Stereotyping, eds Macrae C. N. Stangor C. Hewstone M. (New York, NY: Guilford Press), 254–275.

[B6] Cabinet Administration Office Government of Japan (2014). Opinion Poll on Foreign Diplomacy. Available online at: http://survey.gov-online.go.jp/h26/h26-gaiko/2-1.html

[B7] Couper M. P. (2000). Usability evaluation of computer-assisted survey instruments. Soc. Sci. Comput. Rev. 18, 384–396. 10.1177/089443930001800402

[B8] Couper M. P. Tourangeau R. Conrad F. G. Zhang C. (2013). The design of grids in web surveys. Soc. Sci. Comput. Rev. 31, 322–345. 10.1177/0894439312469865 25258472 PMC4172361

[B9] Darley J. M. Gross P. H. (1983). A hypothesis-confirming bias in labeling effects. J. Pers. Soc. Psychol. 44, 20–33. 10.1037/0022-3514.44.1.20

[B10] Devine P. G. (1989). Stereotypes and prejudice: Their automatic and controlled components. J. Pers. Soc. Psychol. 56, 5–18. 10.1037/0022-3514.56.1.5

[B11] Fiske S. T. Neuberg S. L. (1990). A continuum of impression formation, from category-based to individuating processes: Influences of information and motivation on attention and interpretation. Adv. Exp. Soc. Psychol. 23, 1–74. 10.1016/S0065-2601(08)60317-2

[B12] Garcia-Marques L. Mackie D. M. (1999). The impact of stereotype-incongruent information on perceived group variability and stereotype change. J. Pers. Soc. Psychol. 77, 979–990. 10.1037/0022-3514.77.5.979 10573875

[B13] Göritz A. S. (2004). Recruitment for online access panels. Int. J. Mark. Res. 46, 411–425.

[B14] Göritz A. S. (2006). Incentives in web studies: methodological issues and a review. Int. J. Internet Sci. 1, 58–70.

[B15] Göritz A. S. Wolff H.-G. Goldstein D. G. (2008). Individual payments as a longer-term incentive in online panels. Behav. Res. Methods 40, 1144–1149. 10.3758/BRM.40.4.1144 19001406

[B16] Greszki R. Meyer M. Schoen H. (2014). The impact of speeding on data quality in nonprobability and freshly recruited probability-based online panels, in Online Panel Research: A Data Quality Perspective, eds Callegaro M. Baker R. Bethlehem J. Göritz A. S. Krosnick J. A. Lavrakas P. J. (Chichester: Wiley), 238–262.

[B17] Hainmueller J. Hangartner D. Yamamoto T. (2015). Validating vignette and conjoint survey experiments against real-world behavior. Proc. Natl. Acad. Sci. U.S.A. 112, 2395–2400. 10.1073/pnas.1416587112 25646415 PMC4345583

[B18] Hainmueller J. Hopkins D. J. (2015). The hidden American immigration consensus: a conjoint analysis of attitudes toward immigrants. Am. J. Pol. Sci. 59, 529–548. 10.1111/ajps.12138

[B19] Hauser D. J. Schwarz N. (2015). It's a trap! Instructional manipulation checks prompt systematic thinking on “tricky” tasks. SAGE Open 5, 1–6. 10.1177/2158244015584617

[B20] Hutter R. R. Crisp R. J. Humphreys G. W. Waters G. M. Moffitt G. (2009). The dynamics of category conjunctions. Group Process. Intergroup Relat. 12, 673–686. 10.1177/1368430209337471

[B21] Hutter R. R. Wood C. Turner R. N. (2013). Individuation moderates impressions of conflicting categories for slower processors. Soc. Psychol. 44, 239–247. 10.1027/1864-9335/a000108

[B22] Iyengar S. Jackman S. Messing S. Valentino N. Aalberg T. Duch R. (2013). Do attitudes about immigration predict willingness to admit individual immigrants? A cross-national test of the person-positivity bias. Public Opin. Q. 77, 641–665. 10.1093/poq/nft024

[B23] Kamise Y. Hagiwara S. Lee G. (2010). 2008 Beijing Olympic audience and changes in images of China and Chinese: a panel study of Japanese university students. Keio Media Commun. Res. 60, 67–88.

[B24] Kobayashi T. Collet C. Iyengar S. Hahn K. S. (2015). Who deserves citizenship? An experimental study of Japanese attitudes toward immigrant workers. Soc. Sci. Japan J. 18, 3–22. 10.1093/ssjj/jyu035

[B25] Krosnick J. A. (1991). Response strategies for coping with the cognitive demands of attitude measures in surveys. Appl. Cogn. Psychol. 5, 213–236. 10.1002/acp.2350050305

[B26] Macrae C. N. Milne A. B. Bodenhausen G. V. (1994). Stereotypes as energy-saving devices: a peek inside the cognitive toolbox. J. Pers. Soc. Psychol. 66, 37–47. 10.1037/0022-3514.66.1.37

[B27] Madon S. Guyll M. Aboufadel K. Montiel E. Smith A. Palumbo P. (2001). Ethnic and national stereotypes: the Princeton trilogy revisited and revised. Pers. Soc. Psychol. Bull. 27, 996–1010. 10.1177/0146167201278007

[B28] Mandel D. R. (2014). Do framing effects reveal irrational choice? J. Exp. Psychol. Gen. 143, 1185–1198. 10.1037/a0034207 23978186

[B29] Maniaci M. R. Rogge R. D. (2014). Caring about carelessness: participant inattention and its effects on research. J. Res. Pers. 48, 61–83. 10.1016/j.jrp.2013.09.008

[B30] Ministry of Justice Government of Japan (2015). Statistics of Foreign Residents in Japan As of the End of 2014 (in Japanese). Available online at: http://www.moj.go.jp/nyuukokukanri/kouhou/nyuukokukanri04_00050.html.

[B31] Miura A. Kobayashi T. (2015). Mechanical Japanese: survey satisficing of online panels in Japan. Japanese J. Soc. Psychol. 31, 1–12. 10.14966/jssp.31.1_1

[B32] Miura A. Kobayashi T. (2016). To say, or not to say “Good-bye, Mr./Ms. online survey panels.” J. Media Inf. Commun. 1, 27–42.

[B33] Oppenheimer D. M. Meyvis T. Davidenko N. (2009). Instructional manipulation checks: Detecting satisficing to increase statistical power. J. Exp. Soc. Psychol. 45, 867–872. 10.1016/j.jesp.2009.03.009

[B34] Revilla M. Ochoa C. (2015). What are the links in a web survey among response time, quality, and auto-evaluation of the efforts done? Soc. Sci. Comput. Rev. 33, 97–114. 10.1177/0894439314531214

[B35] Rodin M. J. (1987). Who is memorable to whom: a study of cognitive disregard. Soc. Cogn. 5, 144–165. 10.1521/soco.1987.5.2.144

[B36] Simon H. A. (1957). Models of Man: Social and Rational. New York, NY: Wiley.

[B37] Sporer S. L. (2001). Recognizing faces of other ethnic groups: an integration of theories. Psychol. Public Policy Law 7, 36–97. 10.1037/1076-8971.7.1.36

[B38] Tajfel H. Turner J. C. (1979). An integrative theory of intergroup conflict, in The Psychology of Intergroup Relations, eds Austin W. G. Worchel S. (Monterey, CA: Brooks-Cole), 33–48.

[B39] Tourangeau R. Conrad F. Couper M. (2013). The Science of Web Surveys. New York, NY: Oxford University Press.

[B40] Tsukamoto S. Karasawa M. (2015). From interpersonal to inter-ethnic differentiation: The role of psychological essentialism. J. Hum. Environ. Stud. 13, 13–20. 10.4189/shes.13.13

[B41] Vannete D. L. Krosnick J. A. (2013). Answering questions: a comparison of survey satisficing and mindlessness, in The Wiley Blackwell Handbook of Mindfulness, eds Ie A. Ngnoumen C. T. Langer E. J. (Chichester: John Wiley & Sons, Ltd), 312–327.

[B42] Whitsett H. C. (2013). Understanding Frequent Survey Responders on Online Panels. Nera Economic Consulting, Working Paper. Available online at: http://www.nera.com/publications/archive/2013/understanding-frequent-survey-responders-on-online-panels.html

