# Supplementary material for: Survey Satisficing Inflates Stereotypical Responses in Online Experiment: The Case of Immigration Study
Source: Front Psychol. 2016 Oct 18;7:1563. doi: 10.3389/fpsyg.2016.01563 (PMC5067936; doi:10.3389/fpsyg.2016.01563)
Supplement: Supplementary file 1 [file Image1.pdf]

frontiers

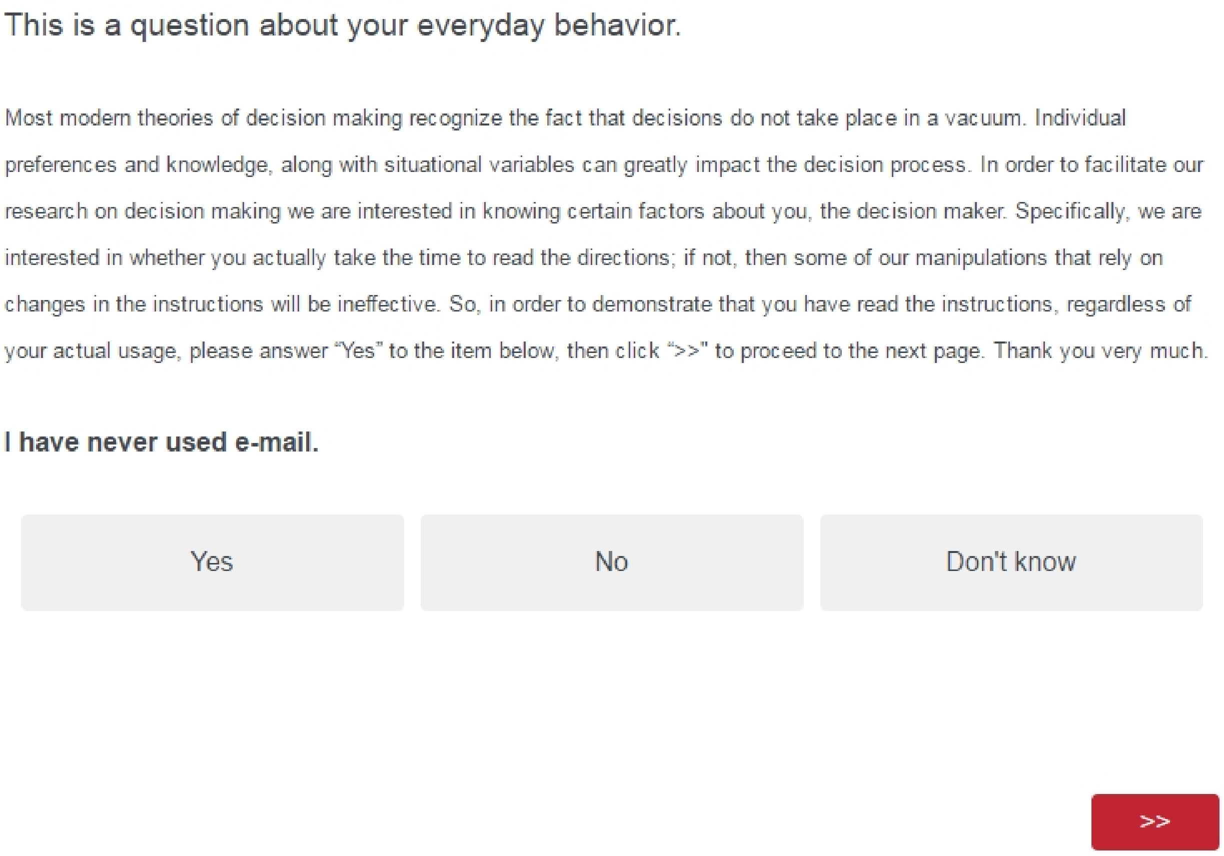

This is a question about your everyday behavior.

Most modern theories of decision making recognize the fact that decisions do not take place in a vacuum. Individual preferences and knowledge, along with situational variables can greatly impact the decision process. In order to facilitate our research on decision making we are interested in knowing certain factors about you, the decision maker. Specifically, we are interested in whether you actually take the time to read the directions; if not, then some of our manipulations that rely on changes in the instructions will be ineffective. So, in order to demonstrate that you have read the instructions, regardless of your actual usage, please answer "Yes" to the item below, then click ">>" to proceed to the next page. Thank you very much.

I have never used e-mail.

Yes

No

Don't know

>>

Supplementary Image 1. Instructional manipulation check
