# Supplementary figures and images for: Survey Satisficing Inflates Stereotypical Responses in Online Experiment: The Case of Immigration Study
Source: Front Psychol. 2016 Oct 18;7:1563. doi: 10.3389/fpsyg.2016.01563 (PMC5067936; doi:10.3389/fpsyg.2016.01563)

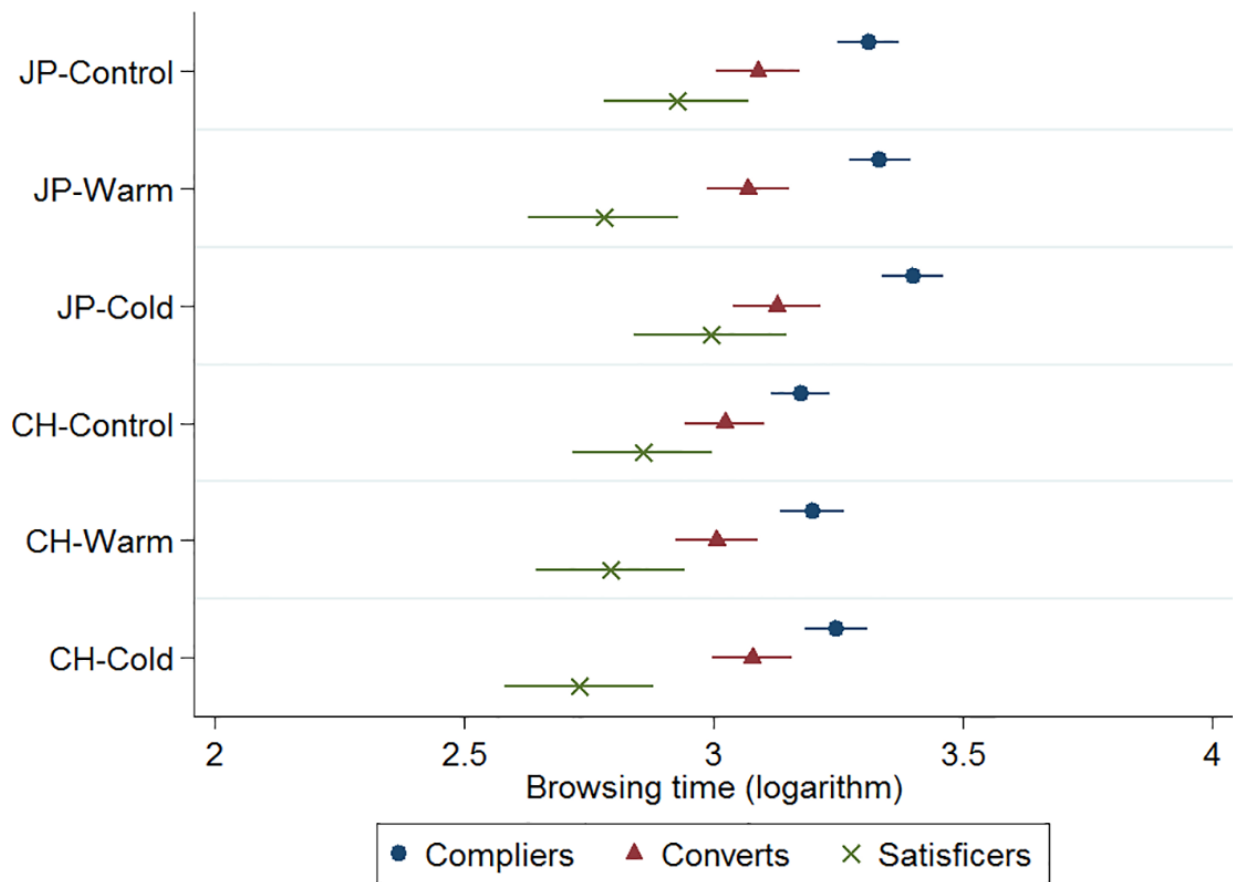

**Supplementary Image 2.** Point estimates on reading time (logarithm) with 95% CI (Study 1).

Supplement: Supplementary file 2 [file Image2.pdf]

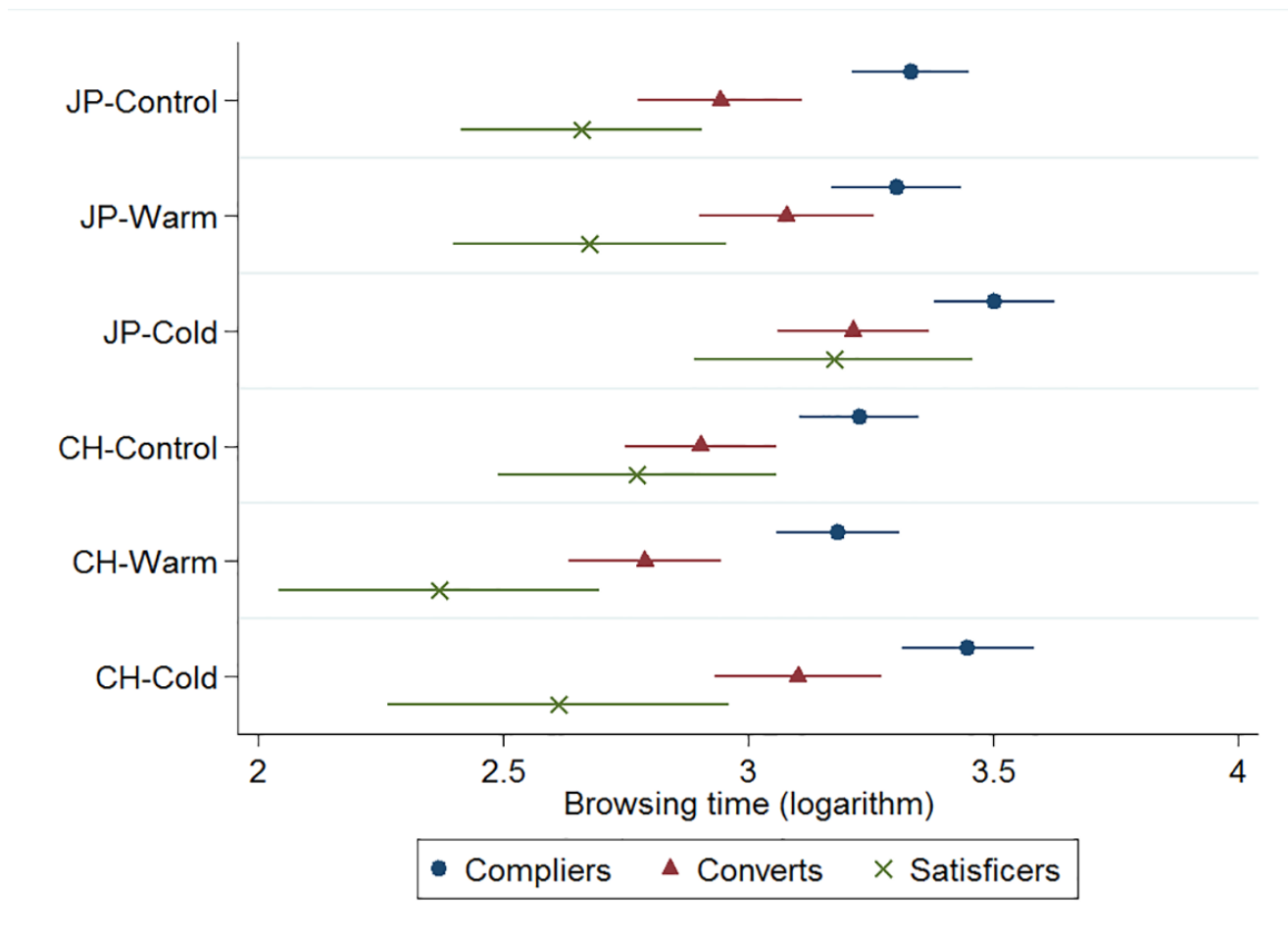

**Supplementary Image 3.** Point estimates on reading time (logarithm) with 95% CI (Study 2).

Supplement: Supplementary file 3 [file Image3.pdf]
