# Supplementary material for: Survey Satisficing Inflates Stereotypical Responses in Online Experiment: The Case of Immigration Study
Source: Front Psychol. 2016 Oct 18;7:1563. doi: 10.3389/fpsyg.2016.01563 (PMC5067936; doi:10.3389/fpsyg.2016.01563)
Supplement: Supplementary file 4 [file Table1.docx]

**Supplementary Table 1.** Bipolar adjectives for rating impressions of target person.

| Considerate of others-Self-centered |
| --- |
| Informal-Formal |
| Sociable-Unsociable |
| Self-assured-Uncertain of himself |
| Popular-Unpopular |
| Humorous-Humorless |
| Important-Insignificant |
| Submissive-Dominant |
| Will go far-Will not get ahead |
| Order of presentation was randomized in each participant. |
