# Supplementary material for: Survey Satisficing Inflates Stereotypical Responses in Online Experiment: The Case of Immigration Study
Source: Front Psychol. 2016 Oct 18;7:1563. doi: 10.3389/fpsyg.2016.01563 (PMC5067936; doi:10.3389/fpsyg.2016.01563)
Supplement: Supplementary file 5 [file Table2.docx]

**Supplementary Table 2.** Ordered logistic regression models predicting satisficing level.

| DV: Satisficing level  (Compliers = 0, Converts = 1, Satisficers = 2) | | Study 1 | Study 2 |
| --- | --- | --- | --- |
|  |  | Coef. (B) | |
| Gender (Baseline: Male) | Female | -0.366** | -0.170 |
|  |  | (0.077) | (0.142) |
| Age |  | 0.012** | 0.016** |
|  |  | (0.003) | (0.006) |
| Marital status (Baseline: Unmarried) | Married | -0.085 | -0.119 |
|  |  | (0.082) | (0.151) |
| Employment status  (Baseline: Other occupations) | Full-time company worker | 0.174* | 0.048 |
|  | (nonsupervisory role) | (0.080) | (0.146) |
|  | Part-time company worker | 0.146 | -0.547* |
|  |  | (0.109) | (0.213) |
|  | Full-time homemaker | -0.043 | -0.169 |
|  |  | (0.112) | (0.205) |
|  | Unemployed | -0.118 | -0.437* |
|  |  | (0.098) | (0.186) |
| Education |  | -0.043 | -0.230+ |
|  |  | (0.036) | (0.119) |
| Device used for survey response  (Baseline: Desktop computer) | Laptop computer | 0.206** | 0.074 |
|  |  | (0.063) | (0.121) |
|  | Mobile device | -0.219* | -0.046 |
|  |  | (0.111) | (0.193) |
| Environment of survey response  (Baseline: Other locations) | Home | -0.074 | -0.089 |
|  |  | (0.088) | (0.159) |
| Frequency of participation  in surveys |  | 0.146** | 0.195** |
|  |  | (0.029) | (0.053) |
| Need for cognition |  | -1.091** | -0.655+ |
|  |  | (0.206) | (0.367) |
| Cut-off point (1) |  | 0.348 | 0.284 |
|  |  | (0.214) | (0.422) |
| Cut-off point (2) |  | 2.279** | 2.233** |
|  |  | (0.217) | (0.429) |
| Number of observations |  | 4,634 | 1,307 |
| LR Chi-squared (13) |  | 159.72** | 45.62** |
| Pseudo *R*^2^ |  | 0.019 | 0.019 |
| Standard errors in parentheses |  |  |  |
| ** *p* < 0.01, * *p* < 0.05, + *p* < 0.1 |  |  |  |
