# Supplementary material for: Survey Satisficing Inflates Stereotypical Responses in Online Experiment: The Case of Immigration Study
Source: Front Psychol. 2016 Oct 18;7:1563. doi: 10.3389/fpsyg.2016.01563 (PMC5067936; doi:10.3389/fpsyg.2016.01563)
Supplement: Supplementary file 6 [file Table3.docx]

**Supplementary Table 3.** Multiple regression models predicting reading time (Logarithm).

| DV:  Reading time (logarithm) |  | Study 1 | Study 2 |
| --- | --- | --- | --- |
|  |  | Coef. (B) | |
| Nationality (Baseline: Japanese) | Chinese | -0.136** | -0.105 |
|  |  | (0.044) | (0.087) |
| Trait (Baseline: Control) | Warm | 0.023 | -0.028 |
|  |  | (0.044) | (0.091) |
|  | Cold | 0.089* | 0.171+ |
|  |  | (0.044) | (0.088) |
| Nationality × Trait (two-way) | Chinese × Warm | 0.001 | -0.014 |
|  |  | (0.063) | (0.127) |
|  | Chinese × Cold | -0.018 | 0.051 |
|  |  | (0.062) | (0.128) |
| Satisficing level  (Baseline: Compliers) | Converts | -0.222** | -0.389** |
|  |  | (0.053) | (0.105) |
|  | Satisficers | -0.385** | -0.673** |
|  |  | (0.080) | (0.139) |
| Nationality  ×  Satisficing level (two-way) | Chinese × Converts | 0.069 | 0.064 |
|  |  | (0.073) | (0.145) |
|  | Chinese × Satisficers | 0.068 | 0.218 |
|  |  | (0.112) | (0.210) |
| Trait  × Satisficing level (two-way) | Warm × Converts | -0.043 | 0.163 |
|  |  | (0.074) | (0.154) |
|  | Warm × Satisficers | -0.170 | 0.045 |
|  |  | (0.115) | (0.210) |
|  | Cold × Converts | -0.050 | 0.100 |
|  |  | (0.076) | (0.145) |
|  | Cold × Satisficers | -0.021 | 0.345 |
|  |  | (0.117) | (0.211) |
| Nationality × Trait × Satisficing level (three-way) | Chiniese × Warm × Converts | 0.003 | -0.234 |
|  |  | (0.105) | (0.210) |
|  | Chiniese × Warm × Satisficers | 0.082 | -0.406 |
|  |  | (0.162) | (0.318) |
|  | Chiniese × Cold × Converts | 0.035 | -0.122 |
|  |  | (0.105) | (0.208) |
|  | Chiniese × Cold × Satisficers | -0.177 | -0.728* |
|  |  | (0.163) | (0.324) |
| Covariates | Feeling thermometer (Chinese) | -0.001 | -0.000 |
|  |  | (0.000) | (0.001) |
|  | Feeling thermometer (Japanese) | 0.004** | 0.007** |
|  |  | (0.001) | (0.001) |
| Constant |  | 3.030** | 2.834** |
|  |  | (0.055) | (0.107) |
| Number of observations |  | 4,650 | 1,308 |
| *R*^2^ |  | 0.069 | 0.145 |
| Standard errors in parentheses |  |  |  |
| ** *p*<0.01, * *p*<0.05, + *p*<0.1 |  |  |  |
