# Supplementary material for: Survey Satisficing Inflates Stereotypical Responses in Online Experiment: The Case of Immigration Study
Source: Front Psychol. 2016 Oct 18;7:1563. doi: 10.3389/fpsyg.2016.01563 (PMC5067936; doi:10.3389/fpsyg.2016.01563)
Supplement: Supplementary file 7 [file DataSheet1.docx]

Supplementary Material

Satisficing and Stereotyping Dance Together: The Case of Immigration Study

Asako Miura*, Tetsuro Kobayashi

*** Correspondence:** Asako Miura: asarin@kwansei.ac.jp

# Supplementary Data

## Measures (Study 1)

### Pretreatment measures

**Gender:** 0 = Male (41.80%), 1 = Female (58.20%).

**Age:** *M* = 49.41, *SD* = 13.86, range: 20–70 years.

**Marital status:** 0 = Unmarried (28.10%), 1 = Married (71.90%).

**Employment status:** Office worker = 22.49%, Housewife = 16.28%, Part-time worker = 10.41%, Other occupations = 36.61%, and Unemployed = 14.21%.

**Level of education:** 1 = Less than college (45.17%), 2 = Some college (54.83%).

**Device used for survey response:** 1 = Desktop computer (37.50%), 2 = Laptop computer (51.26%), 3 = Mobile device, e.g., smartphone or tablet (11.24%).

**Environment of survey response:** 1 = Home (84.91%), 0 = Other locations (15.09%).

**Frequency of participation in surveys:** Weekly average of the frequency of participation in online surveys was measured with a range of 0 to 101 times or more; *M* = 8.54, *SD* = 14.41.

**Need for cognition:** Need for cognition was measured with an 8-item Japanese version of Cacioppo and Petty’s (1982) scale, with responses made on a 5-point scale (Kouyama & Fujihara, 1991). Responses were summed and rescaled from 0 to 1 (*M* = 0.52, *SD* = 0.15, range: 0–1, Cronbach’s alpha = .77).

**Feeling thermometer:** Overall feelings of affinity toward Chinese and Japanese people were measured on a feeling thermometer, yielding a score ranging from 0 to 100. The order of nationality presentation was randomized.

Chinese people: *M* = 29.85, *SD* = 19.84

Japanese people: *M* = 72.50, *SD* = 18.07

### Posttreatment measures

**Impression of the target person:** Impression of the target person was measured using 9 semantic differential items rated on a 5-point scale: Considerate of others–Self-centered, Informal–Formal, Sociable–Unsociable, Self-assured–Uncertain of oneself, Popular–Unpopular, Humorous–Humorless, Important–Insignificant, Submissive–Dominant, and Will go far in life–Will not get ahead in life. Responses were summed and rescaled from 0 to 1 (*M* = 0.54, *SD* = 0.12, range: 0–1, Cronbach’s alpha = 0.83).

## Measures (Study 2)

### Pretreatment measures

Gender: 0 = Male (50.79%), 1 = Female (43.21%).

Age: *M* = 49.28, *SD* = 12.83, range: 20–69 years.

Marital status: 0 = Unmarried (25.27%), 1 = Married (74.73%).

Employment status: Office worker = 23.66%, Housewife = 15.73%, Part-time worker = 11.15%, Other occupations = 36.10%, and Unemployed = 13.36%.

Education: 1 = Less than college (42.00%), 2 = Some college (58.00%).

Device used for survey response: 1 = Desktop computer (36.03%), 2 = Laptop computer (50.76%), 3 = Mobile device, e.g., smartphone or tablet (13.21%).

Environment of survey response: 1 = Home (83.59%), 0 = Other locations (16.41%).

Frequency of participation in surveys: Weekly average of the frequency of participation in online surveys was measured with a range of 0 to 101 times or more; *M* = 8.74, SD = 16.98.

Need for cognition: *M* = 0.52, *SD* = 0.15, range: 0–1, Cronbach’s alpha = 0.79.

Feeling thermometer: Chinese people: *M* = 29.18, *SD* = 18.36, Japanese people: *M* = 73.10, *SD* = 18.30.

### Posttreatment measures

Impression of the target person: *M* = 0.52, *SD* = 0.12, range: 0–1, Cronbach’s alpha = 0.83.Supplementary Material should be uploaded separately on submission. Please include any supplementary data, figures and/or tables.

Supplementary material is not typeset so please ensure that all information is clearly presented, the appropriate caption is included in the file and not in the manuscript, and that the style conforms to the rest of the article.
